# Ineffective Shock Deliveries in a Patient with Ischemic Cardiomyopathy: Shock Vector Matters

**DOI:** 10.19102/icrm.2018.091007

**Published:** 2018-10-15

**Authors:** Enes E. Gul, Usama Boles, Bulent Yildirim

**Affiliations:** ^1^Department of Cardiology, Istanbul Medicine Hospital, Istanbul, Turkey; ^2^Heart and Vascular Center, Mater Private Hospital, Dublin, Ireland

**Keywords:** Implantable cardioverter-defibrillator, ischemic cardiomyopathy, shock delivery, superior vena cava coil

## Abstract

A 56-year-old male who had previously received an implantable cardioverter-defibrillator for primary prevention was admitted to the hospital with frequent shocks. Device interrogation revealed ineffective shock deliveries. Possible explanations for failed treatment are discussed herein.

## Case presentation

**Figure 1:**
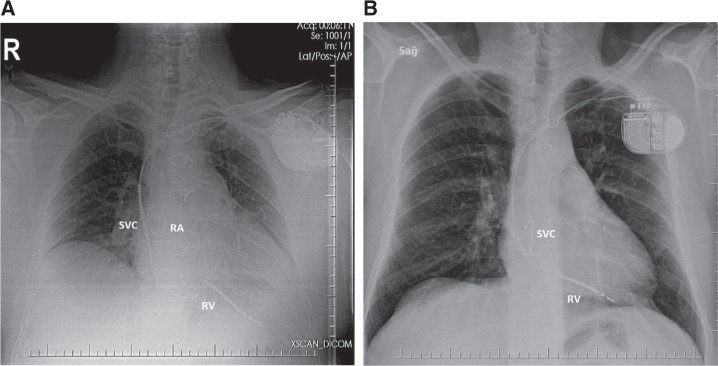
**A:** Postimplantation chest X-ray showing localization of the leads. **B:** In-office chest X-ray showing the SVC coil looped in the RA. SVC: superior vena cava; RA: right atrium; RV: right ventricle.

**Figure 2:**
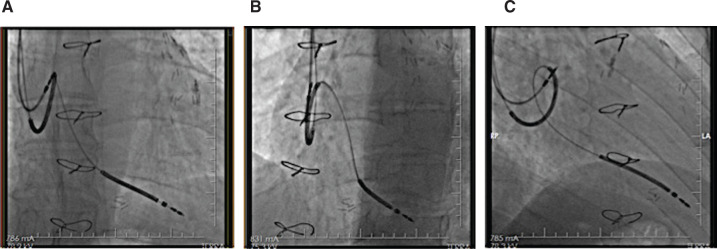
Multiple views of fluoroscopic images confirming localization of the SVC coil. **A:** Anteroposterior view. **B:** Left anterior oblique view. **C:** Right anterior oblique view.

A 56-year-old male with a history of ischemic cardiomyopathy was admitted to the emergency room with frequent episodes of syncope. He had received a dual-chamber implantable cardioverter-defibrillator (ICD) (Iforia-5 DR-T; Biotronik, Berlin, Germany) in 2016 for primary prevention. Device interrogation revealed nine sustained ventricular arrhythmia (VA) episodes and 22 failed shocks. Subsequently, the patient was admitted to the coronary care unit due to electrical storm. Electrocardiogram (ECG) and electrolytes were unremarkable. Device interrogation showed good R-wave sensing at 11.2 mV, a threshold of 0.5 V/0.040 ms, and a shock impedance of 49 Ω. There had been no lead alerts since implantation and the initial shock vector was RV-Can+SVC. Device output during the failed shocks was 40 J (all shock attempts were programmed at the higher energy level of 40 J) and six attempts were made; however, they were unsuccessful. Also, shock impedance was 52 Ω. Further investigation of VA revealed that all episodes were consistent with fast ventricular tachycardia or ventricular fibrillation. Chest X-ray was performed and showed the superior vena cava (SVC) coil looped in the right atrium (RA). This was compared with the postprocedure chest X-ray wherein the SVC coil was originally partially in the SVC, while now it was entirely in the RA near the tricuspid valve **(Figures 1A and 1B)**. The patient was taken to the electrophysiology laboratory and live fluoroscopy was performed in order to figure out the position of the SVC coil. Multiprojectional fluoroscopic images confirmed the SVC coil was present in the RA near the right atrial appendage **(Figure 2)**. The SVC coil was turned OFF. We confirmed the device appropriateness and performed a successful defibrillation test at 10 J. 

In this case, we speculate that the right ventricular (RV) lead migrated towards the RA and RV. It is possible that tricuspid valve movement could have advanced the free-floating SVC coil and, consequently, the RV lead. Failed shock deliveries could be explained with a lack of appropriate shock vectors because of the SVC coil being looped in the RA. After disabling the SVC coil, this issue was resolved. This case highlights the importance of localization of the SVC coil when a dual-coil lead is implanted and shows harm from dual-coil ICD lead implantation. Implantation of single-coil leads should be encouraged in order to prevent such complications and facilitate lead extraction.

